# On-board study of gas embolism in marine turtles caught in bottom trawl fisheries in the Atlantic Ocean

**DOI:** 10.1038/s41598-020-62355-7

**Published:** 2020-03-27

**Authors:** M. L. Parga, J. L. Crespo-Picazo, D. Monteiro, D. García-Párraga, J. A. Hernandez, Y. Swimmer, S. Paz, N. I. Stacy

**Affiliations:** 1 SUBMON Associació, c/Ortigosa 14, 08003 Barcelona, Spain; 2 Research Department. Fundación Oceanogràfic de la Comunidad Valenciana. C/ Gran Vía Marqués del Turia 19, 46005 Valencia, Spain; 3 Projeto Tartarugas no Mar. Núcleo de Educação e Monitoramento Ambiental – NEMA. Rua Maria Araújo, 450, Cassino, Rio Grande, RS 96207-480 Brazil; 4 Department of Large Animal Clinical Sciences, College of Veterinary Medicine, University of Florida, Gainesville, Florida USA; 5 NOAA Fisheries, Pacific Islands Fisheries Science Center, Honolulu, Hawaii USA; 6 Aquatic, Amphibian, and Reptile Pathology Program, Department of Comparative, Diagnostic, and Population Medicine, College of Veterinary Medicine, University of Florida, Gainesville, Florida USA

**Keywords:** Animal physiology, Conservation biology, Environmental impact, Marine biology

## Abstract

Decompression sickness (DCS) was first diagnosed in marine turtles in 2014. After capture in net fisheries, animals typically start showing clinical evidence of DCS hours after being hauled on-board, often dying if untreated. These turtles are normally immediately released without any understanding of subsequent clinical problems or outcome. The objectives of this study were to describe early occurrence and severity of gaseous embolism (GE) and DCS in marine turtles after incidental capture in trawl gear, and to provide estimates of on-board and post-release mortality. Twenty-eight marine turtles were examined on-board fishing vessels. All 20 turtles assessed by ultrasound and/or post-mortem examination developed GE, independent of season, depth and duration of trawl and ascent speed. Gas emboli were obvious by ultrasound within 15 minutes after surfacing and worsened over the course of 2 hours. Blood data were consistent with extreme lactic acidosis, reduced glomerular filtration, and stress. Twelve of 28 (43%) animals died on-board, and 3 of 15 (20%) active turtles released with satellite tags died within 6 days. This is the first empirically-based estimate of on-board and post-release mortality of bycaught marine turtles that has until now been unaccounted for in trawl fisheries not equipped with turtle excluder devices.

## Introduction

Bycatch of marine turtles is a well-documented threat to populations worldwide and, depending on types of fishing gear and nature of interaction, can be associated with high mortality rates^1,2^. Wallace *et al*.^3^ estimated that at least 85,000 marine turtles globally were taken as bycatch in gillnets, longlines, and trawls from 1990 to 2008. In recent years, increased research efforts have focused on understanding population-level impacts of marine turtle bycatch, specifically in trawl and net fisheries^2,4,5^, providing useful information for ecological models, conservation efforts, and fisheries management and policies. However, accurate estimation of mortality remains a persistent challenge, particularly estimates of delayed mortality of turtles that are released from fishing gear alive and later die (i.e., post-release mortality). Satellite telemetry has also emerged as a useful tool for inferring the fate of animals following release^6,7^.

Until recently, mortality of turtles captured in trawl gear has been mainly attributed to the effects of hypoxia and water aspiration associated with forced submergence or to sequelae of traumatic injuries^8–10^. There has been limited study of post-release mortality of marine turtles following capture by trawl fisheries^11,12^. This presents a critical knowledge gap, since such knowledge is essential in informing policy makers to refine sustainable fisheries management practices. In 2014, gas embolism (GE) and consequent decompression sickness (DCS) were first diagnosed in marine turtles captured in trawl and gillnet fisheries in Spain^13^. These novel findings challenged our previous assumptions about marine turtle physiological adaptations to diving^14–16^ and opened a new area of interest regarding the effects of subsurface incidental capture and delayed mortality.

GE refers to gas bubble formation within blood vessels and supersaturated tissues after decompression, whereas DCS is consistent with the clinical diagnosis comprising a wide range of clinical manifestations from gas bubble formation in different tissues^17,18^. DCS is a debilitating and potentially fatal condition that has been reported in marine turtles after alterations of their physiological dive when forcibly retained at depth and then brought to the surface. It is hypothesized that normal pulmonary shunting that takes place during natural dives is reversed during underwater capture, causing increased lung perfusion and nitrogen uptake at depth, resulting in super-saturation and formation of gas emboli in blood vessels and tissues when pressure is reduced during and after ascent^19^.

Although mild GE in marine turtles is usually subclinical, moderate to severe GE typically is associated with various clinical signs of DCS within hours after being hauled on-board due to disrupted blood flow and organ injury by gas bubbles^20^. The most obvious clinical signs are abnormal behavior^13,20^ and neurological alterations, including initial hyperactivity, followed by progressive lethargy, limb paresis, stupor, and loss of sensitivity, rendering the turtle unable to control flipper or head movements or to dive properly. Studies at the Oceanogràfic Sea Turtle Rescue Centre (Valencia, Spain) have shown a prevalence of GE in 52–80% of admitted loggerhead turtles (*Caretta caretta*) bycaught in local trawl and gillnet fisheries^20^, with an estimated 20% of by-caught turtles developing severe GE that likely leads to death by DCS over the course of hours or days if untreated.

Using routine fishing and handling practices, these turtles would likely be released immediately without any understanding of subsequent clinical problems or outcome^21,22^. Therefore, it is necessary to improve our knowledge of the occurrence and severity of DCS in order to accurately inform estimates of marine turtle mortality resulting from bycatch in fisheries, to better understand the role of fishery parameters and environmental factors that may influence the development and progression of DCS, and to identify possible mitigation measures^20^. This information will assist managers and industry to better predict risk of GE and DCS in marine turtles and ultimately to identify means to maximize turtles’ probability of survival after interactions with fishing gear.

The objectives of this study were (1) to describe the occurrence and severity of GE using on-board clinical, ultrasonographical, and blood biochemical assessments of marine turtles during the first 2 hours after incidental capture in trawl gear, and (2) to provide estimates of on-board and short-term post-release mortality. The findings herein enhance our knowledge of this recently discovered phenomenon, expand documented occurrences of DCS outside of the Mediterranean where it was initially observed, and present further evidence that DCS is a largely cryptic cause of fisheries-associated mortality resulting from forced submersion.

## Results

### Trawl conditions

Trawl duration and depth, and ambient temperature did not differ significantly between Groups A and B (see Table 1). Details of trawl depth and duration for each captured individual are presented in Supporting Table S1. Ascent speed was not different (*p* = 0.47) between the groups, but was significantly faster (*p* < 0.01) for turtles captured in winter as compared to those captured during the summer-autumn.

**Table 1:** Comparison of fishing data between marine turtles that were released and survived 30 days (Group A) or died on deck or were released but died post-release (Group B).

Parameter	All Turtles	Group A	Group B	*p*^a^
Ambient temp **°**C	18.5 (14.0, 30.0) n = 14	18.5 (15.0, 30.0) n = 6	21.0 (14.0, 29.0) n = 8	0.87
Curved carapace length (cm)	76 (56, 88) n = 26	76.6 (68.4, 87.0) n = 11	72.5 (56.0, 88.0) n = 15	0.15
Duration trawl (min)	262 (184, 302) n = 26	258 (184, 302) n = 11	262 (210, 300) n = 15	0.39
Depth trawl (m)	45.0 (18.0, 81.0) n = 25	58.0 (18.0, 80.0) n = 11	41.5 (19, 8) n = 14	0.59
Fish in net (kg)	1800 (175, 3500) n = 25	1200 (800, 2800) n = 11	2200 (175, 3500) n = 14	0.26
Time from surfacing to first exam (min)	24 (9, 375) n = 26	17 (9, 375) n = 11	25 (10, 75) n = 15	0.40
Ascent speed (m/min); all	2.1 (0.9, 12.0) n = 23	2.2 (1.2, 3.4) n = 10	1.7 (0.9, 12.0) n = 13	0.47
Ascent speed (m/min); winter	3.0 (1.7, 12.0) n = 14	3.2 (2.0, 3.4) n = 7	3.0 (1.7, 12.0) n = 7	0.97
Ascent speed (m/min); summer	1.5 (0.9, 1.9) n = 9	1.5 (1.2, 1.9) n = 3	1.5 (0.9, 1.6) n = 6	0.76

Data are reported as median (minimum, maximum).

^a^*p*-values indicate comparison of data between Groups A and B.

### Study animals

Of 28 turtles, 14 were incidentally captured during summer-autumn and 14 during winter (Supporting Table S1). Most of the study turtles were large juvenile or adult loggerheads (CCL range 62.5 cm–88 cm), except for 1 adult olive ridley (CCL 71.9 cm). All animals were in good body condition. Seven turtles had fresh superficial abrasions on neck and flippers and 2 had acute cloacal prolapse, likely caused during the trawl capture. Twelve turtles arrived alert and active (n = 5 summer-autumn, n = 7 in winter) and were released with sPAT tags. Five turtles arrived weak (n = 2 in summer-autumn, n = 3 in winter), of which 2 died on-board and 3 improved over the course of 2 hours on deck, and were released with sPAT tags. Eleven turtles were very weak or minimally responsive (e.g., blink response) but alive upon arrival (n = 7 in summer-autumn, n = 4 in winter); of these, 10 died on-board (n = 6 in summer-autumn; n = 4 in winter), while only 1 improved over time and was released several hours later without a sPAT tag.

### Ultrasound examination

Ultrasonography was performed during 1 summer and 1 winter trip due to limited equipment availability. In total, 16 turtles were scanned; 8 during each trip. Initial scans were conducted within 45 minutes of surfacing for 14 of these turtles; the remaining 2 turtles were initially scanned at 70 and 277 minutes, respectively. The turtle that was scanned at 277 minutes arrived at the pair-vessel and could not be examined until 4.5 hours later after transfer to the main vessel, thus, this animal was scanned to confirm GE but excluded in the clinical and post-release mortality study – please see Supporting Information. Approximately 15 minutes post-surfacing was the earliest scans could be performed due to logistical constraints. GE was confirmed in all animals during the initial scan and was consistently found in the kidney and renal vessels. It was not feasible to evaluate the heart or major vessels of most turtles due to their large size. The amount of GE in tissues and blood vessels increased with time during the course of the 2-hour monitoring period. The final GE severity scores reached during the period of evaluation are shown in Table 2. Although there were no significant differences between Groups A and B (*p* = 0.58), or between summer and winter captures (*p* = 0.08), GE scores were more variable among turtles captured during winter.

**Table 2:** Comparison of gas embolism (GE) severity scores after 2 hours on a scale of 1–3 between marine turtles that were released and survived 30 days (Group A) or died on deck or were released but died post-release (Group B).

	All	Group A	Group B	*p*^b^
GE severity score, total	3.0 (1.0, 3.0) n = 15	2.0 (2.0, 3.0) n = 5	3.0 (1.0, 3.0) n = 10	0.58
GE severity score, winter	2.0 (1.0, 3.0) n = 8	2.0 (2.0, 3.0) n = 3	2.0 (1.0, 3.0) n = 5	0.67
GE severity score, summer	3.0 (2.0, 3.0) n = 7	2.5 (2.0, 3.0) n = 2	3.0 (3.0, 3.0) n = 5	0.28

Data are reported as median (minimum, maximum).

^b^*p*-values indicate comparison of data between Groups A and B.

### Blood analysis

PCV was higher in turtles of Group A (median = 48%) compared to those in Group B (37%) (*p* ≤ 0.04), and phosphorus and potassium concentrations were lower in Group A (median = 2.8 mmol/L and 3.7 mmol/L, respectively) compared to Group B (3.4 mmol/L and 5.5 mmol/L, respectively) (*p* ≤ 0.05) (Table 3).

**Table 3:** Comparison of packed cell volume and plasma chemistry data upon arrival on boat deck between marine turtles that were released and survived 30 days (Group A) or died on deck or were released but died post-release (Group B).

Analyte	All (min, max)	Group A (min, max)	Group B (min, max)	*p*^c^
Packed Cell Volume (PCV) spun %	40.0 (29.0, 48.0) n = 14	48.0 (41.0, 48.0) n = 5	37.0 (29.0, 45.0) n = 9	<0.01
Calcium mmol/L	1.9 (0.6, 3.5) n = 14	1.8 (0.6, 2.5) n = 6	2.0 (1.4, 3.5) n = 8	0.34
Phosphorus mmol/L	3.2 (2.5, 5.5) n = 14	2.8 (2.5, 4.0) n = 6	3.4 (2.9, 5.5) n = 8	0.05
Magnesium mmol/L	2.1 (1.5, 2.9) n = 14	2.1 (1.5, 2.9) n = 6	2.0 (1.5, 2.9) n = 8	0.68
Sodium mmol/L	155.0 (142.0, 169.0) n = 14	151.0 (144.0, 162.0) n = 6	158.0 (142.0, 169.0) n = 8	0.29
Potassium mmol/L	4.8 (3.3, 6.5) n = 14	3.7 (3.3, 5.4) n = 6	5.5 (3.7, 6.5) n = 8	0.02
Chloride mmol/L	129 (113, 144) n = 11	129 (113, 144) n = 5	127 (113, 135) n = 6	0.99
Blood Urea Nitrogen mmol/L	38.5 (4.2, 104.9) n = 14	30.1 (4.2, 76.0) n = 6	54.9 (20.8, 104.9) n = 8	0.22
Lactic acid mmol/L	27.4 (13.4, 60.4) n = 14	24.1 (13.4, 44.7) n = 6	34.0 (18.4, 60.4) n = 8	0.49
Creatinine umol/L	17.6 (12.3, 44.2) n = 14	17.6 (14.1, 44.2) n = 6	16.7 (12.3, 17.6) n = 8	0.61
Glucose mmol/L	9.2 (2.8, 18.4) n = 14	8.7 (2.8, 12.8) n = 6	9.6 (3.5, 18.4) n = 8	0.41
Cholesterol mmol/L	4.4 (1.7, 7.2) n = 14	4.7 (1.8, 7.2) n = 6	4.0 (1.7, 6.2) n = 8	0.36
Triglycerides mmol/L	1.7 (0.3, 5.2) n = 14	1.5 (0.4, 3.5) n = 6	1.7 (0.3, 5.2) n = 8	0.99
Uric acid umol/L	142.7 (41.6, 344.9) n = 14	127.8 (41.6, 344.9) n = 6	145.7 (53.5, 285.5) n = 8	0.63
Aspartate Transaminase (AST) ukat/L	3.5 (1.7, 5.4) n = 14	3.6 (2.6, 4.2) n = 6	2.6 (1.7, 5.4) n = 8	0.57
Alanine Aminotransferase ukat/L	0.10 (0.05, 0.30) n = 8	0.12 (0.05, 0.20) n = 4	0.18 (0.06, 0.34) n = 4	0.45
Lactate Dehydrogenase ukat/L	3.7 (0.9, 35.8) n = 14	4.4 (1.1, 11.2) n = 6	3.7 (0.9, 35.8) n = 8	0.66
Creatine Kinase ukat/L	17.5 (9.7, 34.2) n = 13	19.0 (9.7, 34.2) n = 5	17.3 (15.1, 19.9) n = 8	0.72
Total protein g/L	46.5 (28.0, 58.0) n = 14	47.0 (33.0, 57.0) n = 6	41.0 (28.0, 58.0) n = 8	0.59
Albumin (protein electrophoresis) g/L	1.7 (0.8, 4.3) n = 13	2.6 (0.8, 3.7) n = 6	1.5 (1.0, 4.3) n = 7	0.70
Albumin:Globulin ratio	0.5 (0.1, 1.4) n = 13	0.6 (0.1, 0.8) n = 6	0.4 (0.2, 1.4) n = 7	0.59
Alpha 1-globulins g/L	0.3 (0.2, 4.0) n = 13	0.2 (0.2, 1.9) n = 6	0.4 (0.2, 4.0) n = 7	0.36
Alpha 2-globulins g/L	13.1 (8.9, 18.0) n = 13	13.2 (11.3, 14.7) n = 6	11.8 (8.9, 18.0) n = 7	0.80
Beta 1-globulins g/L	11.0 (3.4, 16.9) n = 13	12.0 (3.5, 13.4) n = 6	10.3 (3.4, 16.9) n = 7	0.62
Beta 2-globulins g/L	8.7 (2.9, 26.2) n = 13	7.3 (3.4, 26.2) N = 6	9.2 (2.9, 22.4) n = 7	0.83
Gamma-globulins g/L	8.9 (0.3, 27.5) n = 13	13.2 (0.3, 18.1) n = 6	1.0 (0.5, 27.5) n = 7	0.62

Data are reported as median (minimum, maximum).

^c^*p*-values indicate comparison of data between Groups A and B.

### Neurological examination

Ten of 15 examined animals of Group B had minimal or no response to reflex and response evaluation upon boarding. In comparison, 11 of 12 animals of Group A (*p* < 0.01) were responsive to stimuli, although their responses were highly variable among individuals and over time. Four animals of Group A and 1 of Group B were considered more hyper-responsive/aggressive in their reactions after 1 hour on-board, e.g., immediately opening their mouth and trying to bite when the neck was touched or abruptly removing flippers when contacted.

### On-board mortality

In total, 12 (43%) of 28 captured animals died on-board. Based on necropsy examinations, all turtles that died had severe GE based on the amount of observed gas emboli and anatomical distribution^13^. Intravascular gas bubbles were immediately obvious in peripheral small blood vessels after opening the plastron and easily visible in large amounts in the cardiac atria, sinus venosus, and pulmonary, mesenteric, and splenic veins.

Six of 7 animals that died during the summer-autumn trips also had evidence of water aspiration, including tracheal and bronchial oedema (n = 5) or large amounts of fluid in the lungs (n = 6). In winter, 2 of 5 necropsied animals had oedema or fluid in the respiratory airways.

### Post-release mortality

Sixteen animals survived and were considered fit for release. 15 of these were equipped with sPAT tags (6 in summer and 9 in winter). Three of the 15 tagged animals (20%) died during the first 6 days; they initially arrived weak (moved slowly and only when touched) but seemed to improve clinically before release. The outcome of a fourth animal is unknown. The deployed tags did not provide information on the diving profile of tagged turtles, but all live animals dove to a maximum depth of 9 to 138 meters at least once every day during the tracking period.

## Discussion

This is the first study to document the occurrence, severity, and progression of GE in marine turtles captured by commercial bottom trawl gear and to provide estimates of on-board and post-release mortality of turtles released without treatment. This study is also the first report of GE in marine turtles outside the Mediterranean Sea, indicating a high likelihood that DCS is a global concern among all fisheries that capture turtles in a manner that results in underwater entrapment or forced submergence. Furthermore, GE was diagnosed in an olive ridley sea turtle for the first time. To date, four species of marine turtles, including loggerhead, green, leatherback, and olive ridley have been diagnosed with GE^13^, (Garcia-Párraga & Crespo-Picazo pers. com.).

All turtles assessed by ultrasonography and/or post-mortem examination developed various degrees of GE. GE developed immediately and was detectable in all cases at the time of initial assessment, as soon as 15 minutes post-surfacing. We did not expect such a high frequency of occurrence based on previous studies in Spain, which found GE in only 43–55% of turtles captured by trawl at depths of 25–75 meters^13,20^. Notably, turtles examined during these earlier studies were evaluated up to 8–10 hours post-capture. It is possible that those with mild GE were able to eliminate and resolve the gas bubbles prior to examination. Our understanding of the progression and resolution of GE under natural conditions, as well as the various factors that may influence this condition and its clinical outcome, remains limited and worthy of further study^20^.

The severity of GE quickly worsened over the 2-hour monitoring period, with most animals reaching a 3/3 severity score, particularly those caught during the warmer months. This progression was less evident in animals caught during the winter, although seasonal differences were not statistically significant (p = 0.08) and there was no association between season and turtle mortality rates. Sasso and Epperly^9^ reported a higher mortality rate of turtles captured by trawling in colder months, thus we were interested as to whether such a seasonal affect could be associated with risk of GE. We did not find evidence of this, but our sample size was small. Various factors, such as prior dives, activity level, or previous fishery interactions may influence gas bubble formation in marine turtles and confound interpretation of results, especially when sample sizes are limited, as is often the case due to many logistical challenges associated with fisheries-based studies^23^.

The post-release mortality rate for turtles examined in our study was 20% based on the satellite telemetry results for 15 bycaught turtles, 11 of which survived at least 30 days. This rate is most relevant to those turtles that are active and alert upon release, and did not include animals that were weak, but responsive and subsequently died on-board, which may have been immediately released if not for our study. Additional mortality beyond 30 days from long-term effects of GE needs to be considered, but was not assessed in this study. There are in fact other potential health risks associated with development of GE that may cause a long-term mortality^18,24^. Although further studies of GE and long-term mortality are warranted, accurate determination of post-release mortality is logistically challenging, especially over 90 days after release, due to the numerous factors that can influence risk of death^6^.

Our mortality rate is in contrast to findings by Maxwell *et al*.^12^, who reported that 3 comatose bycaught turtles revived on-board vessels survived at least 30 days after release. In their study, however, trawl depth was shallower and of shorter duration compared to our study (i.e., 12 and 17 meters, and 1,5 to 3,5 hours, respectively) and presence of GE was not assessed. Most unresponsive turtles encountered during our study died and were found to have both GE and evidence of water aspiration. In our experience, GE and DCS entail a worse prognosis if they occur concurrently with water aspiration^13,25^.

All turtles but two (captured during the cold season) developed moderate to severe GE as measured by ultrasonography. While this degree of GE in turtles admitted to rehabilitation centres tends to be fatal when untreated^13^, nearly 80% of released turtles in our study survived at least for 30 days post release. One possible explanation for the lower mortality in our study is that turtles may have been in the early gas-off phase with intravascular gas forming micro-bubbles throughout the body, leading to an overestimated GE scoring by ultrasound. However, in animals admitted to rehabilitation centres several hours after the decompression event, the gas-off phase is most probably over. Most circulating gas emboli may have already been eliminated through the lung via respiration, and the residual gas tends to be retained in organs and tissues, forming larger nitrogen bubbles after coalescence. This results in a much lower degree of GE at that point, even when originally and based only on ultrasonography it would have been scored as more severe. Moderate and severe GE cases seen at rehabilitation centres are those cases that accumulate so much gas from the onset of cardiorespiratory failure that clearance is hindered during the first 8–12 hours after surfacing.

Another hypothesis is that, once released, active turtles may be able to dive back to depths necessary to recompress, resulting in reduction of bubble size and allowing partial reperfusion and re-dissolution of nitrogen bubbles formed after surfacing. Repeated diving and ascent phases coupled with minimal time at the surface may allow turtles to successfully manage elimination of non-dissolved nitrogen bubbles over several days. This could minimize the risk of accumulating large amounts of bubbles in circulation, which would otherwise impede normal tissue or organ perfusion. In this study, the diving profile of released animals could not be assessed, but records of the transmitters show that all animals dived between 9 to 138 meters at least once every day. To confirm this hypothesis, it would be useful to evaluate the diving profile of decompressed turtles during the first days after release and compare their behaviour with healthy turtles. Based on maximum registered diving depths recorded by sPAT tags of the study animals, we believe that deeper dives did not occur immediately after the decompression event.

Results of clinical evaluations were similar to those previously described with DCS in loggerheads^25,26^. Compared to blood data from clinically normal marine turtles^27,28^, blood analyses indicated physiological and hemodynamic derangements with reduced glomerular filtration rate attributable to forced submergence, physical exertion, and GE. The degree of lactic acidosis was extreme and up to 3 times higher than reported for loggerhead turtles caught by trawl at shallower depths using shorter tows of 30 minutes^29^. We also found evidence of reduced renal function that was more pronounced in turtles that died as suggested by significantly higher potassium and phosphorus concentrations. GE is known for its main accumulation around the kidney region with time^13^. Furthermore, PCV was significantly lower in turtles that died, suggesting potential impaired blood flow, shunting, or shock, given the context of lactic acidosis. Tissue enzyme activities (e.g., creatine kinase, aspartate aminotransferase, lactate dehydrogenase, and alanine aminotransferase) upon arrival on deck were similar to stranded loggerhead sea turtles reported in Deem *et al*.^27^, and slightly above normal ranges for foraging turtles, suggesting effects from trawling and/or developing GE.

Although blood data from turtles at time of release was not available, the observed clinical and ultrasonographical presence and worsening of GE while on-board would presumptively result in continuous development of tissue damage while on board. This, in addition to comparatively higher glucose, demonstrates the presence of anaerobic metabolism resulting from hypoventilation and reduced tissue perfusion from forced submergence, in addition to stress. Shorter trawl periods would likely be conducive to reduce these detrimental pathophysiological effects.

The neurological examination yielded highly variable and inconsistent responses and was not considered clinically useful in the evaluation of GE or its severity. In 5 animals, the response was considered “hyper-responsive”, with the animal immediately trying to bite or abruptly removing limbs when touched. Such responses are consistent with previously reported findings in marine turtles with GE in Spain^25,26^ and are believed to be indicative of severe pain caused by nitrogen bubbles accumulating in different tissues.

Our observations highlight some significant challenges in caring for bycaught turtles in a manner that maximizes their survival post-release. We documented multiple well-known effects of underwater capture, including severe physiological derangements and risk of seawater aspiration. These conditions benefit from opportunity for recovery following capture and expelling water from the airways, as incorporated into common handling practices and recommendations for “resuscitation” of bycaught marine turtles^30^. On the other hand, we also documented a high frequency of GE in conjunction with clinical indications of DCS and resulting mortality, conditions that may be exacerbated when decompressed turtles remain at the surface without treatment. These findings demonstrate that the effects of underwater capture on marine turtles are complex and have clinically significant implications that are not easily addressed under fishing conditions. For all these reasons, the authors emphasize the importance of integrating the use of TEDs into all trawling fisheries with a high capture-rate of marine turtles as a legal requirement, since TEDs are the only way to significantly reduce incidental captures. However, we acknowledge the fact that it may take years before TEDs become a legal requirement in different parts of the world. Until TED implementation into fishing regulations will come into effect, the following summarizes our recommendations for trawlers not carrying TEDs, reflecting our current understanding and assessment:

- Until further research is completed under different trawl conditions, assume that GE occurs in all turtles captured by trawls lasting more than 3 hours and greater than 17 m of depth regardless of ambient temperature. It should be emphasized that the presence of GE is not always associated with clinically overt manifestation of DCS;
- Active turtles captured under the above conditions may benefit from being released as soon as possible to give them the highest chance of recovery. Keeping bycaught turtles on-board allows for continuation of gas bubble formation, presumptively worsening its pathophysiological effects. At this time it is not possible to determine how long a turtle can remain on-board safely;
- If a turtle is unresponsive or weak and in need of time to recuperate, it is imperative to place it in a cool/shaded area for recovery. Warm temperatures may increase the rate of GE, likely worsening its effects;
- The only situation in which it is advisable to retain a turtle on-board for a few hours, regardless of its clinical condition (active vs. unresponsive), is for transport to a qualified rehabilitation centre with means of adequate treatment, such as hyperbaric chamber or alternative specific GE/DCS treatment options. Treatment increases the probability of survival to almost 100%^26^. Otherwise, based on current knowledge, we strongly recommend releasing turtles back to the sea as soon as they are active and alert, and when conditions are safe to do so.

Gas embolism associated with trawl fisheries was first diagnosed in marine turtles in 2014^13^ and there is still very little known about its incidence in turtles captured by different types of fisheries around the world, its progression over the first hours post-capture, and its resulting acute and delayed effects and mortality. Further research is imperative to ascertain how fisheries operational parameters and environmental conditions affect the onset and severity of GE in marine turtles, and to develop further recommendations to reduce DCS-related mortality. Another essential field of research is the behaviour and outcome of released turtles in order to improve recommendations for immediate handling of incidentally captured turtles in trawl and other net fisheries.

## Methods

No turtles were specifically caught or sacrificed for the purpose of this study. This study was designed by veterinarians specialized in marine turtles and carried out only by trained veterinarians and marine biologists. Blood sampling and ultrasonography were performed by veterinarians. Handling and sampling protocols were based on NMFS-SEFSC 2008 Sea Turtle Research Techniques Manual^30^. All experiments were performed in accordance with relevant guidelines and regulations. The study was carried out under research permit 15962-6 and 15962-7 issued by the Brazilian government.

### Trawl fishery

This work was carried out in collaboration with a bottom pair trawl fishery operating off the coast of Rio Grande do Sul, Southern Brazil, described in Table 4. In this type of fishery, two boats simultaneously tow along a single net along the seafloor. Fishing occurs day and night and all year round. Main target species are Argentine croaker (*Umbrina canosai*), stripped weakfish (*Cynoscion guatucupa*), white croaker (*Micropogonias furnieri*) and Southern king weakfish (*Macrodon atricauda*). Incidental capture of marine turtles occurs throughout the year, with a range of 2–15 animals per trip. Loggerhead sea turtles are most frequently captured, although green turtles (*Chelonia mydas*), olive ridley (*Lepidochelys olivacea*) and leatherback sea turtles (*Dermochelys coriacea*) are also occasionally captured^31^. Turtle Excluder Devices (TEDs) are not used and are not required by law in Brazil.

**Table 4:** Technical information of the bottom pair trawl fishery of Southern Brazil used in this study.

Parameter	Data for single operation (2 vessels)
Length	16.9–27 m
Engine power	167–360 hp.
Loading capacity	50–160 t
Crew	6–7 fishers
Net dimension: main body	12–40 m
Net dimension: end sac	12–29 m
Net dimension: stretched mesh size	80–150 mm
Trawl depth	15–90 m
Towing time	3–7 h
Towing speed	2–3.5 knots

We completed 6 fishing trips, 4 during austral summer-autumn (January and April 2017, February and April 2019) and 2 during austral winter (July-August and September 2018). For each trawl with a captured turtle, we reported fishing parameters, (e.g., trawl depth and duration), speed at which the net was hauled up to the vessel (i.e., ascent speed), kg of fish captured, and ambient temperature.

### Clinical assessment of marine turtles

Clinical evaluations were initiated as soon as a turtle was accessible on deck after net recovery, and included routine general physical examination, measurement of curved carapace length (CCL), neurological examination, ultrasonography [General Electric Logiq E Vet 5.2x machine with linear (12LRS) and microconvex (8CRS) probes], and blood sample collection for packed cell volume (PCV) and plasma biochemical analysis. Animals were kept on-board on a tire and monitored for 2 hours before release.

We performed an initial ultrasound scan to assess the presence of GE in tissues and blood vessels. This was achieved by confirming the presence of hyper-echoic foci and comet tail artefacts in renal vessels, portal-renal vein, and heart and associated major vessels, compatible with intravascular gas emboli^13^. We followed Valente *et al*.^32^ to access the renal and cardiac regions. We repeated ultrasound scans of the right kidney, heart, and major blood vessels every 30–40 minutes for 2 hours. After 2 hours, we scored the severity of GE based on ultrasonographical findings of each turtle as mild (1), moderate (2) or severe (3) following García-Párraga *et al*.^13^.

Immediately after initial ultrasonography, we used a 10 ml syringe and a 21 G 60 mm hypodermic needle to aseptically collect whole blood from the dorsal cervical sinus. Blood was collected with lithium-heparin anticoagulant and analysed for PCV and plasma biochemical analytes using standard methods (Laboratório Antonello; Pelotas, Rio Grande do Sul, Brazil).

A routine physical examination followed to assess the general condition of each turtle and to identify any clinical signs related to incidental capture (e.g., visible wounds, evidence of entanglement or water aspiration). We then performed a basic neurological examination based on Chrisman *et al*.^33^ and repeated it every 30 minutes until the release, if the turtle remained alive. This examination included assessment of general activity level, head posture, bite response, palpebral reflex, neck retraction when touched, flexor reflexes of all 4 flippers, superficial dermal nociception of flippers, and cloacal reflex. If the animal was unresponsive, we administered manual resuscitation measures as often as possible between examinations, following general guidelines^29^. No special medications or medical equipment were taken on board; therefore, resuscitation measures were limited to physical measures: placing the animal in ventral recumbency with elevation of both hindquarters to allow water draining from the lungs, and keeping the temperature of the animal similar to the temperature of sea water and the skin moistened. These resuscitation measures were demonstrated and recommended to fishermen for future use.

### Post-release satellite telemetry

Survivorship Pop-up Archival (sPAT) tag (Wildlife Computers, Redmond WA, USA) were attached to live and active turtles before release, using an adaptation of the technique described by Maxwell *et al*.^12^. We drilled a 2 mm diameter hole in the margin of the turtle’s left or right supracaudal scute and tightly clamped two 1 mm nylon brackets between the carapace and the tag to reduce the chance of accidental entanglements. Animals were released immediately after the tag was placed. Only one turtle, which arrived at the pair-vessel and could not be examined until 4.5 hours later, was excluded from this study and was released without a sPAT tag (see Supporting Information).

All tags activated by contact with seawater and operated for 30 days. On day 30, tags released by an automated mechanism, floated to the surface, and transmitted the collected data. If there was any evidence that the turtle had died before the 30-day limit, the tag was released from the animal via a corrodible pin. Mortality criteria were defined as: (1) tag floats continuously for 24 h (floater); or (2) tag sinks to the bottom and remains at constant depth for 24 h (sinker). Information transmitted by the tags included GPS location at release, survivorship status of the animal, and daily maximum and minimum water temperatures and depth.

### Post-mortem examinations

Animals that appeared dead (e.g., absence of responses to evaluation of reflexes in 30 to 40 minute intervals for more than 3 hours) were left on deck until onset of *rigor mortis* in jaw was confirmed. At that time, resuscitation measures were discontinued and we performed an on-board necropsy to confirm GE. We positioned the carcass in dorsal recumbency on a car tire and carefully removed the plastron, minimizing artifactual gas infiltration. First, we incised the pectoral muscles and the pericardial sac to inspect the heart, blood vessels, and deep muscle for gas emboli^13^. Next, we conducted an initial inspection of the coelomic cavity to detect gas emboli within mesenteric blood vessels. Following these steps, we opened the entire coelomic cavity to evaluate organs for emboli, lesions, or disease. The respiratory system was specifically examined for froth and fluid indicative of water aspiration.

### Data analysis

We divided the study turtles into Group A (released and survived 30 days) and Group B (died on deck or post-release). We used the Wilcoxon Rank Sum test to assess the null hypotheses that the distribution of fishing parameters, GE severity scores from turtles captured in winter or summer, and blood chemistry data were not different between Groups A and B. We also tested the null hypothesis that ascent speed was not different between marine turtles captured in winter or summer by using the Wilcoxon rank sum test. Finally, we investigated potential differences in the frequency of turtles classified as aggressive, non-responsive, reflexes present, or with an abnormal neurological examination by using a chi-square test. Values of *p* ≤ 0.05 were considered statistically significant. All analyses were conducted using Statistix 10 for Windows (Analytical Software, Tallahassee, Florida).

## Supplementary information

Supplementary information.

## Data Availability

A table summarizing fishing conditions and clinical findings for each turtle (Appendix S1) is available online. All data are available on request to the corresponding author.
