# Supplementary material for: On-board study of gas embolism in marine turtles caught in bottom trawl fisheries in the Atlantic Ocean
Source: Sci Rep. 2020 Mar 27;10:5561. doi: 10.1038/s41598-020-62355-7 (PMC7101392; doi:10.1038/s41598-020-62355-7)
Supplement: Supplementary file 1 — Supplementary information. [file 41598_2020_62355_MOESM1_ESM.pdf]

On-board study of gas embolism in marine turtles caught in bottom trawl fisheries in the Atlantic Ocean

Parga, M.L. [1]*, Crespo-Picazo, J.L.[2], Monteiro, D.[3], García-Párraga, D.[2], Hernandez, J.A.[4], Swimmer, Y.[5], Paz, S.[3], Stacy, N.I.[6]

1. SUBMON Associació, c/Ortigosa 14, 08003 Barcelona, Spain

2. Research Department. Fundación Oceanogràfic de la Comunidad Valenciana. C/ Gran Vía Marqués del Turia 19, 46005. Valencia, Spain.

3. Projeto Tartarugas no Mar. Núcleo de Educação e Monitoramento Ambiental – NEMA. Rua Maria Araújo, 450, Cassino, Rio Grande – RS, Brazil, 96207-480.

4. Department of Large Animal Clinical Sciences, College of Veterinary Medicine, University of Florida, Gainesville, Florida, USA

5. NOAA Fisheries, Pacific Islands Fisheries Science Center, Honolulu, Hawaii, USA

6. Aquatic, Amphibian, and Reptile Pathology Program, Department of Comparative, Diagnostic, and Population Medicine, College of Veterinary Medicine, University of Florida, Gainesville, Florida, USA

Supplemental Table 1: Summary of trawl conditions, time from surfacing to exam, physical exam findings, presence of gaseous emboli (GE), and outcome of each captured marine turtle.

Trip #	Time of year	Turtle ID	Sp[a]	CCL[b] (cm)	Depth of trawl (m)	Length of trawl	Kg of fish in net	Time from net on surface to first exam	Physical exam on arrival	Presence of GE[c] confirmed?	Outcome onboard	Outcome post-release
1	Summer	CC17/001	Cc[d]	76.3	20	4h25m	1,400	25min	Good body condition. No external injuries. Comatose.	Yes (US[e] scan and PM[f])	Died on board: necropsy	-
		CC17/002	Cc	93.5	17	4h	2,345	4h37min (brought to other boat)	Good body condition. No external injuries. Comatose.	Yes (US scan[g])	Improved while on board. Released after 5 h without tag	-
		CC17/003	Cc	72.5	20	4h22m	2,800	19min	Good body condition. No external injuries. Comatose.	Yes (US scan and PM)	Died on board: necropsy	-
		CC17/004	Cc	70.6	20	4h22m	2,800	28min	Good body condition. Some fresh scratches. Bright and active	Yes (US scan)	Released after 2h40m with tag.	Survived for 30 days
		CC17/005	Cc	67.6	20	4h22m	2,800	34min	Good body condition. Fresh cloacal prolapse. Comatose.	Yes (US scan and PM)	Died on board: necropsy	-
		CC17/006	Cc	78.5	20	4h22m	2,800	44min	Good body condition. No external injuries. Comatose.	Yes (US scan and PM)	Died on board: necropsy	-
		LO17/007	Lo[h]	71.9	19	4h33m	175	29min	Good body condition. Fresh scratches, ray's sting in skin and cloacal prolapse. Comatose.	Yes (US scan and PM)	Died on board: necropsy	-
		CC17/008	Cc	83.9	18	3h6m	1,050	15min	Good body condition. Fresh wounds in flipper, large subcutaneous hemorrhage in flipper. Bright and active	Yes (US scan)	Released after 2h28m with tag.	Survived for 30 days
2		CC17/010	Cc	83	48	4h37m	7,000	Unknown	Good body condition. No external injuries. Bright and active.	No US scan	Released after 1h50m with tag.	Unknown
		CC17/012	Cc	76	27	4h45m	1,050	6h15min (brought to other boat)	Good body condition. No external injuries. Bright and active.	No US scan	Released after 5h with tag.	Survived for 30 days
3	Winter	CC18/001	Cc	87	66	3h46m	1,200	1h10min (only seen after 1 hour on deck, under the fish)	Good body condition. No external injuries. Bright and active.	Yes (US scan)	Released after 2h5m with tag.	Survived for 30 days

		CC18/002	Cc	80	80	3h56m	1,200	15min	Good body condition. Some fresh scratches. Bright and active.	Yes (US scan)	Released after 2h10m with tag.	Survived for 30 days
		CC18/003	Cc	83	78	4h	1,400	39min	Good body condition. No external injuries. Bright and active.	Yes (US scan)	Released after 2h25m with tag.	Survived for 30 days
		CC18/004	Cc	66.3	60	4h		16min	Good body condition. No external injuries. Weak on arrival.	Yes (US scan)	Improved while on board. Released after 2h5m with tag.	Floater after 6 days
		CC18/005	Cc	77.7	60	4h33m	2,400	35min	Good body condition. No external injuries. Weak on arrival.	Yes (US scan)	Improved while on board. Released after 2h10m with tag.	Sinker after 1day
		CC18/006	Cc	68	38	3h55m	1,000	39min	Good body condition. No external injuries. Comatose.	Yes (US scan and PM)	Died on board: necropsy	-
		CC18/007	Cc	81.1	81	4h54m	2,500	38min	Good body condition. No external injuries. Comatose.	Yes (US scan and PM)	Died on board: necropsy	-
		CC18/008	Cc	62.5	45	4h23m	1,700	21min	Good body condition. Scratches in flippers. Arrives weak, and soon worsens.	Yes (US scan and PM)	Died on board: necropsy	-
4		CC18/009	Cc	72.1	60.4	4h50m	2,000	17min	Good body conditions. No external injuries. Bright and active.	No US scan	Released after 2h2m with tag.	Survived for 30 days
		CC18/010	Cc		60,4	4h50m	2,000	17min	Good body conditions. No external injuries. Extremely weak.	Yes (PM)	Died on board: necropsy	
		CC18/011	Cc	76.6	58	4h18m	2,500	9min	Good body conditions. No external injuries. Bright and active.	No US scan	Released after 2h with tag.	Survived for 30 days
		CC18/012	Cc	68.4	60	4h26m	800	25min	Animal captured by the other boat and transferred immediately. Good body conditions. No external injuries. Bright and active.	No US scan	Released after 2h with tag.	Survived for 30 days
		CC18/013	Cc	74	55	4h11m	700	23min	Animal captured by the other boat and transferred immediately. Good body condition. No external injuries. Comatose.	Yes (PM)	Died on board: necropsy	
		CC18/014	Cc	80	36.5	5h02m	1,200	13min	Good body conditions. No external injuries. Bright and active.	No US scan	Released after 2h6m with tag.	Survived for 4 months (tag malfunctioned and only got released and reported after 4 months)
5	Summer	CC19/001	Cc	76	26	3h4m	2,500	17min	Good body conditions. Superficial abrasions in shoulders. Bright and active.	No US scan	Released after 2h with tag.	Survived for 30 days
		CC19/002	Cc	71	38	4h19m	3,500	10min	Good body condition. No external injuries. Arrives weak.	Yes (PM)	Died on board; necropsy	
6		CC19/003	Cc	83.8	45	3h30m	1,800	17min	Good body conditions. No external injuries. Weak on arrival, improving with time.	No US scan	Released after 2h with tag.	Sinker after 6 days
		CC19/004	Cc	88	37	5h	2,500	75min	Good body conditions. No external injuries. Comatose.	Yes (PM)	Died on board; necropsy	

[a] Sp = species
[b] CCL = Curved carapace length
[c] GE = Gaseous embolism
[d] Cc = *Caretta caretta*
[e] US = Ultrasound examination

[f] PM = Post-mortem examination
[g] US only for confirmation of GE; this individual was excluded from US progression study
[h] Lo = *Lepidochelys olivacea*
